# Systematic Review on Psychological Impacts and Mitigating Strategies of COVID-19: The Lesson Every One Shall Learn From the Our Time Pandemic

**DOI:** 10.1177/10541373211005110

**Published:** 2022-10

**Authors:** Awgchew Shimelash Yasegnal

**Affiliations:** 1 Department of Psychology, College of Education and Behavioral Sciences, Bahir Dar University, Bahir Dar, Ethiopia

**Keywords:** COVID-19, psychological impacts, mitigating strategies, worsening factors, every one, our time pandemic

## Abstract

**Objective:**

The pandemic is expanding exponentially, burning, and threatening the world population regardless of differences. Cognizant of these, world nations make it their daily agenda and give due concern in preventing and intervening it. However, the prevention and intervention strategies are of more biological and less attention is given for the psychological impacts. So that this manuscript is intended to review the psychological impacts and mitigating strategies of COVID-19 in Ethiopia.

**Method:**

Out the 63 downloaded articles, 26 articles were selected by considering relatedness, reputable journal, and pattern of writing and reviewed.

**Results:**

Most reviewed studies reported negative psychological impacts including, boredom, loneliness, anger, violence and abuse, distress, low mood and irritability, anxiety, insomnia, hopelessness and worthless, and suicide. Other associated factors like poverty, fears of infection, duration of quarantine, lack of genuine and adequate information, lack of basic supplies, stigma, housing condition, and cultural issues potentially worsen the psychological impacts.

**Conclusion:**

Psychological intervention strategies like mobilizing volunteers and professionals, identifying vulnerable population, assuring psychological readiness, offering adequate and genuine information, providing adequate supplies, improving communication for those in quarantine, utilizing counseling platform and rehabilitation program are of the cures for psychological impacts of COVID-19.

**Introduction**

These days, the COVID-19 outbreak has become one of the largest crises appearing all over the world. The world population is sick both biologically and psychologically within the outbreak of the pandemic. Following this largest pandemic, governments of the world-wide nations have implemented preventive and intervention strategies like self-isolation, quarantine, physical distancing, wearing face masks, keeping personal hygiene, and staying at home. Though the governments in the world are using those strategies, the virus has profoundly affected daily life of the people throughout the world (Lades et al., 2020).

The exponential, rapid, and largely uncontrolled spread of COVID-19 has influenced every facet of world population’s life, calling for dramatic shifts in the social and professional behavior of the people (Jacobson et al., 2020). The preventive strategies and the battles against COVID-19 have worsened the usual life of the world-wide population. Population’s adherence to the governments’ guidelines on both preventive and intervention strategies is essential that will more likely decrease the biological impacts of the current pandemic (Zhong et al., 2020).

Although physical distancing, self-isolation, quarantine, and staying at home measures are necessary to protect and treat biological health, less is known about the impact of such measures on the people’s psychological well-being (Jacobson et al., 2020). The prospect of becoming physically unwell with COVID-19 ranked lower than these issues related to the psychological problems due to the pandemic (Holmes et al., 2020).

Working from home, loss of employment, and social and physical distancing have abruptly interrupted many social opportunities important to physical and psychological health. It is important to research and learn from successful existing strategies to keep up and build social resources and resilience and promote good psychological well-being in the populations moving forward (Holmes et al., 2020). People's emotional responses during massive infectious disease outbreaks are likely to include feelings of extreme fear and uncertainty that, along with being separated from loved ones and the limitations on freedom, may eventually lead to dramatic psychological problem (Brooks et al., 2020).

In Ethiopia, the first case of the pandemic was reported on March 3, 2020, and the prevalence now is becoming devastating and it will worsen the life of the citizens if preventive and intervention strategies are not efficiently employed with the consideration psychological crisis. Following the report of the first incidence, the government in the state established a National Ministerial Committee on 16 March 2020. As the concern is mounting, the government and the parliament declared a state of emergency on April 8–10, 2020, and the council of ministers issued regulation on 11 April, 2020.

The National Ministerial Committee among others emphasizes prevention and protection; a 14 days mandatory quarantine of passengers arriving to Ethiopia, avoiding public gathering, health sector capacity building, regulating market to avoid unethical exploitation of the situation; declaring state of emergency, and supporting regions’ preparedness to prevent the disease have been implemented. Similarly, the emergency proclamations and the regulation among others emphasizes avoiding handshakes, reducing the number of public transport passengers to 50%, keeping adequate physical distancing, providing cleaning and hand washing facilities in each public institutions. Furthermore, some civil service institutions were closed (e.g. higher education institutions, primary and secondary schools) while some others were operating with less than 50% workforce, suggesting public services were substantially reduced (Debela, 2020).

Unlike most western countries of the world, Ethiopians’ community psychology is largely collective culture. Unfortunately, the preventive and intervention strategies of COVID-19 are of strictly individualistic, which are the direct converse of the indigenous psychological makeup of the nations, nationalities and Peoples in Ethiopia. It is therefore, for most of the Ethiopian citizens, preventive and intervention measures are means of psychological crises.

To date, in Ethiopia, epidemiological data on the mental health problems and psychiatric morbidity of those suspected or diagnosed with the COVID-19 and their treating health professionals have not been available; so that how best to respond to psychological challenges during and after the outbreak is unknown. Discussing and disclosing the psychological impacts of COVID-19 alone will never be a relief for the society; indicating and also implementing ways of overcoming its psychological impacts is a vital rather.

## Psychological Impacts of COVID-19

The sudden outbreak of a disease always poses the threat to the psychological wellbeing of affected people and their close contacts. Confirmed patients, suspected patients, and medical and related people who haveclose contacts with patients may have the possibility of having a higher prevalence of anxiety, depression, anger, and other associated psychological problems. Patients may have the fear of death, doctors and nurses those are involved in treating COVID-19 affected people may experience a fear of contagion by this virus and spreading to their family, friends, or close others (Xiang et al., 2020).

Patients with confirmed or suspected with COVID-19 may experience fear of the consequences of infection with a potentially fatal new virus, and those in quarantine might experience boredom, loneliness, and anger. Furthermore, symptoms of the infection, such as fever, hypoxia, and cough, as well as adverse effects of treatment, such as insomnia caused by corticosteroids, could lead to worsening anxiety and mental distress (Xiang et al., 2020).

These days, most people are intensively worrying about being of infected and infecting others as well. Studies reported that fears about their own health or fears of infecting others are high among people during such pandemics (Bai et al., 2004; Cava et al., 2005; Desclaux et al., 2017; Hawryluck et al., 2004).People became particularly worried if they experienced any physical symptoms potentially related to the infection (Desclaux et al., 2017).

The confinement, the loss of the habitual routine and the reduction of the social contact are some of the most distressing factors for the people in quarantine, which end up generating frustration and irritability (Brooks et al., 2020).

During the COVID-19 emergency, people are afraid of infection/being infected and infecting others/, dying, and losing family members. Women and children have also experienced increased domestic violence and abuse (Jacobson et al., 2020). Widespread misinformation about the virus and prevention measures and deep uncertainty about the future are other major sources of distress. Repeated media images of severely ill people, dead bodies and coffins add to the fear. People may not have the opportunity to say goodbye to dying loved ones and may not be able to hold funerals. Cognizant of these incidents, people further develop distress (Ahmed et al., 2020).

Measures used to combat COVID-19 were associated with higher levels of psychological distress including post-traumatic stress symptoms, confusion and anger, and high prevalence of low mood and irritability (Brooks et al., 2020; Holmes et al., 2020; Jacobson et al., 2020), and this implies that clear action shall be taken that may cut psychological distress (Wang et al., 2011). Research results show that topics related to anxiety (i.e. fear, anxiety, avoiding, restlessness, procrastination), negative thoughts about oneself and the future (i.e. hopelessness and worthless), sleep disturbances (i.e. insomnia), and suicidal ideation (i.e. suicide, suicidal) were each associated with dramatic increases prior to stay-at-home orders being announced with next and considerable leveling off during these periods, about the same time as the stay-at-home orders were announced and enacted (Jacobson et al., 2020).

Patients who survived severe and life-threatening illness were at risk of post-traumatic stress disorder and depression. Many of the anticipated consequences of quarantine and associated social and physical distancing measures are themselves key risk factors for psychological issues. These include suicide and self-harm, alcohol and substance misuse, gambling, domestic and child abuse, and psychosocial risks (such as social disconnection, lack of meaning or anomie, entrapment, cyber bullying, feeling a burden, financial stress, bereavement, loss, unemployment, homelessness, and relationship breakdown) (Holmes et al., 2020).

Furthermore, research in china revealed that the prevalence of psychological problems associated with people’s incarceration to the COVID-19 epidemic is high. Much higher rate of anxiety, depression, alcohol consumptions, and lower mental wellbeing among Chinese people were found due to COVID-19 outbreak and their conﬁnement in their home as the first-line response to the epidemic or public health emergency (Ahmed et al., 2020).

Despite the common psychological problems found among people, patients, and health workers during such outbreaks, most health professionals working in isolation units and hospitals do not receive any training in providing psychological interventions. And hence, timely psychological intervention strategies need to be developed and implemented urgently.

However, before applying the mitigating strategies, know how on the other associated factors which will worsen the COVID-19’s psychological impact is very important. This is aimed at answering the question *“what will worsen the psychological problems during COVID-19 outbreak?”* While applying measures like lockdown, quarantine, staying at home, and physical distancing, parallel problems will arise that in turn aggravate the psychological problems. To optimize effectiveness of psychological treatments, triggering factors which are casually associated with poor psychological wellbeing shall get due concern before an intervention is taken (Holmes et al., 2018). I here, therefore, list out certain factors that could aggravate its psychological effects in Ethiopia:

## What Other Associated Factors Worsen the Psychological Impacts of COVID-19?

**Poverty:** In low and middle-income countries without social safety nets, the effects on population health and health inequalities are likely to be worse than in richer countries (Douglas et al., 2020). Notwithstanding the excellent (but a bit exaggerated) growth recorded in Ethiopia in the last decade and half, the majority of Ethiopians live a precarious and poverty-stricken life. Using the official poverty rate of 22 percent, about 26 million of the estimated 108 million population of the country lives below poverty line. This is an understated figure because it is computed using Birr 20 per day per adult (that is a salary of Birr 600 per month) as poverty line. It is obvious that it is difficult to live with this money in today’s Ethiopia (Geda, 2020).

Apart from this general picture of poverty, the labor force in the country is predominantly engaged in the precarious and informal sectors. Such people are extremely vulnerable to partial lock-down measure that break markets and disrupt food and essential goods supply chains or raise their prices. The profile of the Ethiopian urban labor market shows how vulnerable the most of urban population is to the economic effect of partial lock-down and social-distancing policies that will aggravate frustration and emotional disturbances. This is because even those who could work during this time earns, on average, only 3,132 Birr per month. This means the majority leads a hand-to mouth existence for which staying at home entails hunger (and eventually starvation) sooner or later, perhaps after a month or so (Geda, 2020).

• **Duration of Quarantine:** Quarantines of more than 10 days usually cause more negative emotional states and their psychological repercussions are greater (Hawryluck et al., 2004). However, these days, 14 days of quarantine is becoming mandatory considering the incubation period of the virus which will worsen the psychological well-being of the persons in quarantine. An extension of quarantine, however small, can greatly exacerbate frustration. Longer durations of quarantine were associated with poorer psychological well-being specifically, post-traumatic stress symptoms (Hawryluck et al., 2004), avoidance behaviors, and anger (Marjanovic et al., 2007). Being isolated creates a defenseless situation that is very difficult to manage.

• **Lack of Genuine and Adequate Information:** Information is vital to create awareness and make people stable about with the nature of the pandemic. However, due to lack of technological advancement and the nature of the people’s settlement in Ethiopia, adequate information is not provided for the population. Even information from the unknown sources misleads people to develop wrong conception about the pandemic. If we do not have reliable information, catastrophic thoughts skyrocket and we fall into a dangerous loop of negativity (Brooks et al., 2020). Perceived lack of transparency from health and government officials about the severity of the pandemic and the lack of clear guidelines or rationale, perceived difficulty with complying with quarantine protocols was a significant predictor of post-traumatic stress symptoms (Reynolds et al., 2008).

During this critical time, the role of effective and prompt communication to educate people, to share information, and to change the behavior of citizens is significant. However, it would be difficult to reach all citizens in Ethiopia since the huge majority of people do not have access to the internet and media (radio and television).

• **Lack of Basic Supplies, Such as Food, Personal Protective Equipment and Medicines:** Since the outbreak of the pandemic, most manufacturing organizations have been reduced their workforces which in turn lowers the production of basic supplies. Lack of personal protective equipment for health professionals, laboratory supplies for examining the virus (masks, gloves and thermometers) absence of medicines for the infected persons, and food for quarantined patients are seriously influencing the psychological states of the people. During a period of pandemic, people cannot give themselves the basic things they need; so lacking them or not having a regular supply further sharpens the feeling of lack of control and worsening psychological problems.

On all accounts, the institutional capacity of the Ethiopian Health Institution is weak even when compared to the Sub-Saharan African average. The inadequate number and the quality of medical staff is also a critical concern. Concerning the quantity, according to the World Bank database, the proportion of physicians (per 1,000 people), and nurses and midwives (per 1,000 people), in 2017, in Ethiopia was only 0.1 and 0.84 respectively (Debela, 2020). Most medical laboratories and research institutes in the world and in Ethiopia now are trying their best to invent treatment drugs and vaccine if possible though clues of treatment drugs and vaccine haven’t get proved as of yet.

**Stigma:** In a comparison of health-care workers quarantined versus those not quarantined, quarantined participants were much more likely to report stigmatization and rejection from people in their local neighborhoods (Brooks et al., 2020). Participants/people in quarantine/also reported that others were treating them differently: avoiding them, withdrawing social invitations, treating them with fear and suspicion, and making critical comments (Cava et al., 2005).

**Housing Condition:** Most urban people in Ethiopia are living through rents of dormitories which are not scattered, with in slums, and on streets. This implies that, staying at home strategy is distressing. Having such living conditions, controlling the outbreak of the virus would be extremely challenging.

**Cultural Issues:** Ethiopians are collective society; the society gives more weight to group well-being than personal freedom. This cultural context may have negative influences on preventing the virus because preventive and intervention strategies of COVID-19 are of individualistic; applying those strategies may result separation anxiety, adjustment disorder, and depression among the society.

The collective culture can open ground for the spectacular spread of the virus. Partly due to cultural values, the society may not comply with health professionals’ and official prescriptions and advice. The research conducted by Debela (2020) revealed that during the first period, citizens were not complying with the advice of health professionals; they were going to religious institutions. This cultural context could discourage committed civil servants from providing services on one hand and increase their vulnerability to the diseases on the other.

Knowing those associated factors listed above, another issue should also be answered; what to do so? Developing and implementing the mitigating strategies of the impacts of COVID-19 is the remaining crucial thing.

## Mitigating Strategies

The lacks of psychological support systems and the lack of well-trained psychiatrists and/or psychologists added to the other associated factors increased the risks of psychological maladjustment (Shultz et al., 2015). During the outbreak, mental health professionals and psychologists should actively take part in the overall intervention process for the disease, so that the psychological responses could be integrated in a timely fashion (Mohammed et al., 2015). Crucially, reducing sustained feelings of loneliness and promoting belongingness are candidate mechanisms to protect against, anxiety, depression, insomnia, suicide, self-harm, and impulsiveness.

According to Zhang et al. (2020), psychological crisis interventions should be integrated into the treatment and blocking of the transmission routes. Psychological crisis intervention should include two simultaneous activities: (1) intervention for fear of disease, carried out mainly by physicians and assisted by psychologists; (2) intervention for difficulty in adaptation, mainly by social psychologists. Among them, serious mental problems (e.g. violence, suicide behaviors) shall be managed by psychiatrists.

The Preventive and intervention measures of COVID-19 together with the associated factors have the above listed psychological impacts; but the preventive and intervention measures are of necessary because there are no effective means of treatment for the virus as of yet. Hence, mitigating the psychological impact of such measures is invaluable and the only alternative that the world countries have at hand now. I therefore, here below, summarize tailored strategies which are supposed to be effective in mitigating the psychological impacts of COVID-19 in Ethiopia.

**Mobilizing Volunteers and Concerned Professionals:** Psychologists, psychiatrists, social workers, sociologists, and community volunteer members shall be selected and organized in the form of task force for the battle against psychological problems.

**Identifying Vulnerable Population Groups for Due Intervention:** Though the virus will potentially attack all populations regardless of differences, infected persons, front line health professionals, and people with other related diseases, elders, street population … etc are highly vulnerable to the pandemic. Health workers who come in close contact with the virus and are exposed to traumatic events, such as death and dying, while making highly challenging decisions, are particularly at risk of stress responses (Li et al., 2020). During the COVID-19 pandemic, it is important that health and social care workers are supported to stay in work and hence psychological treatment approaches are likely to be a key part to discuss complex psychological conditions, coping mechanisms, and prevention (Holmes et al., 2020).

Health care workers who have worked directly with the confirmed patients in the hospitals have the likelihood to suffer from physical and psychological problems. Consequently, it is important to keep them updated of the treatment process along with the psychosocial training because precise knowledge reduces the risk of psychological problems. Healthcare workers are also going through a kind of quarantine that can lead to high levels of depression, anxiety, and post traumatic stress syndrome, in addition to their high occupational stresses. For this reason, healthcare workers should be included in the treatment and training plan (Ahmed et al., 2020).

**Assuring Psychological Readiness:** Psychological preparation on the events matters on the side effects of incidents. Centuries ago, Seneca, the Roman philosopher, stated that *“the unexpected has more overwhelming effects, adding to the weight of the disaster.”*

**Providing Adequate and Genuine Information**: Information will both kill and cure population. If people are exposed to rumors and propaganda, they will develop psychological problems which in the long run potentially bring the death of many. Rumors induced fear while an epidemic outbreak underestimates the coping strategy of people and this underestimation created panic. This panic gets potency with the uncertainty of COVID-19. The repeated broadcast through the media about the fatality of COVID-19 could trigger a sense of jeopardy. There is the risk of delayed psychological trauma and anxiety even after recovering from the epidemic. That is why (Ahmed et al., 2020) recommend that staying connected to reliable news media and not focusing on the absurd misleading information on social media.

On the other hand, information will make light sever psychological problems. Given the unique circumstances of COVID-19, digital psychological interventions that are mechanistically informed, along better understanding of the buffering effects of social relationships during stressful events, are required (Brooks et al., 2020).

Digital interventions for psychological problems include awareness creation, information provision, automated and blended therapeutic interventions (such as apps and online programs), telephone calls and messages to reach those with poorer digital resources (digital poverty). Looking beyond digital interventions (as not everyone has access to them), and ascertaining what other mechanistically based psychological interventions are effective and for whom is important (Holmes et al., 2020).

Using internet access, Mass Medias (TV and radio), Magazines, Journals, and Home to home ways of information provision are methods to be used to lower psychological problem. However, in the context of Ethiopia, more than 80% of the population lives in rural area with no internet, TV, radio, magazines, and journal accesses. Hence, *community based home to home awareness creation* shall also be conducted by volunteers and professionals.

**Providing Adequate Supplies:** Fulfilling Personal protective equipment for health professionals will decrease their frustration of being infected and infecting others as well. If possible, provision of housing for those professionals engaged in treating patients with COVID-19 is the best way to cutoff the transmission of the virus to the families of health professionals and this is an invaluable strategy to cut anxiety, depression, frustration, stigma, and insomnia that the professionals, their family members, and the neighborhood members will face. Officials also need to make sure that quarantined households have enough supplies for their basic needs and importantly, these shall be provided as rapidly as possible.

**Reducing the Boredom and Improve the Communication for Those in Quarantine:** Boredom and isolation will cause distress; people who are quarantined should be advised about what they can do to stave off boredom and provided with practical advice on adjustment skills, coping skills,and stress management techniques. Having a working mobile phone is now a necessity, not a luxury, and those stepping off a long flight to enter quarantine will probably welcome a charger or adaptor more than anything else (Jeong et al., 2016).

Having a telephone support line, staffed by psychiatric nurses and psychologists, and set up specifically for those in quarantine could be effective in terms of providing them with a social network (Maunder et al., 2003). The ability to communicate with one’s family and friends is also essential. Particularly, social media could play an important part in communication with those far away, allowing people who are quarantined to update their loved ones about their situation and reassure them that they are well. In so doing, people in quarantine will develop sense of belongingness and connected; feeling connected is essential to deal with quarantine. Activating our social network through phone calls or social networks allow people to keep up contact and not feel so alone.

Therefore, providing those quarantined with mobile phones, cords and outlets for charging devices, and robust Wi-Fi networks with internet access to allow them to communicate directly with loved ones could cut feelings of isolation, stress, and panic (Manuell & Cukor, 2011).

Although this is possible to do in enforced quarantine, it could be more difficult to do in the case of widespread home quarantine; countries imposing censors on social media and messaging applications could also present difficulties in ensuring lines of communication between those quarantined and their loved ones.

It is also important that public health officials keep up clear lines of communication with people quarantined about what to do if they experience any symptoms. A phone line or online service specifically set up for those in quarantine and staffed by health-care workers who can give instructions about what to do in the event of developing illness symptoms would help to reassure people that they will be cared for if they become ill. This service would show those who are quarantined that they have not been forgotten and that their health needs are just as important as those of the wider public. Such deeds then decrease feelings such as fear, worry, and anger (Brooks et al., 2020).

Psychological follow-up for those in quarantine shall focus on finding and developing an altruistic sense. In fact, the psychological impact of a chosen quarantine will be much less than that of mandatory isolation. It is, therefore, to make sense of what is happening to them and understand that isolation is helping to keep others safe, including the people they love, but also those particularly vulnerable. It is also about being aware and responsible.

**Utilizing Counseling Platform:** Now to help the problematic groups, the government of Ethiopia needs to be more conscious to mobilize the counseling platforms. Higher education institutions need to work with their University Counseling Centers (UCC) to offer counseling services through online, Mass Medias/radios and televisions/, and or face to face counseling/with maintaining the required physical distance/. Issues like self-psychological adjustment skills, emotional stability, how to face the anxiety and fear, how to lower insomnia, and relaxation trainings shall be given.

**Figure 1.**
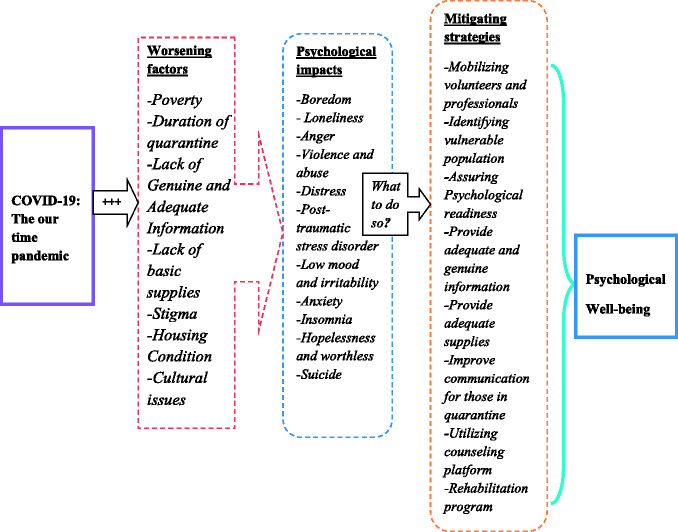
A Model Summary of Psychological Impacts and Mitigating Strategies of COVID-19 in Ethiopia.

**Launching Rehabilitation Program:** The 21st century has seen the corona virus epidemic twice before the outbreak of COVID 19. Hence, the findings and learning of SARS-CoV and MERS-CoV could be applied for the COVID-19 epidemic. Discharged patients of SARS-CoV from hospitals have developed anxiety, depression, and PTS syndrome (Lee et al., 2007) and after a while it turns into neurological and neuromuscular symptoms and both patients and healthcare workers are vulnerable collaterally (Ahmed et al., 2020). So, it is important that long-term psychological support and rehabilitation program shall be available for sustained recovery (Figure 1).

The following diagram attempts to summarize the psychological impacts and mitigating strategies of COVID-19:

## Conclusion

This review affirmed that the psychological impacts of the preventive and intervention measures of the current pandemic, COVID-19 are of substantial and may be enduring. However, avoiding those measures is unthinkable since other means of treatments haven’t confirmed as of yet. Moreover, associated factors like Poverty, fears of infection, duration of quarantine, lack of genuine and adequate information, lack of basic supplies, stigma, housing condition, and cultural issues potentially worsen the psychological impacts. Psychological impacts like boredom, loneliness, anger, violence and abuse, distress, low mood and irritability, anxiety, insomnia, hopelessness and worthless, suicide, and post-traumatic stress disorder are common. Therefore, psychological intervention strategies like mobilizing volunteers and professionals, identifying vulnerable population, assuring psychological readiness, offering adequate and genuine information, providing adequate supplies, improving communication for those in quarantine, utilizing counseling platform, and launching rehabilitation program are the cures of psychological impacts of COVID-19.
